# Gut Microbiome and Space Travelers’ Health: State of the Art and Possible Pro/Prebiotic Strategies for Long-Term Space Missions

**DOI:** 10.3389/fphys.2020.553929

**Published:** 2020-09-08

**Authors:** Silvia Turroni, Marciane Magnani, Pukar KC, Philippe Lesnik, Hubert Vidal, Martina Heer

**Affiliations:** ^1^Unit of Microbial Ecology of Health, Department of Pharmacy and Biotechnology, University of Bologna, Bologna, Italy; ^2^Laboratory of Microbial Processes in Foods, Department of Food Engineering, Federal University of Paraíba, João Pessoa, Brazil; ^3^Institut National de la Santé et de la Recherche Médicale (Inserm, UMR_S 1166), Hôpital de la Pitié-Salpêtrière, Sorbonne Université, Paris, France; ^4^Institute of Cardiometabolism and Nutrition, Hôpital Pitié-Salpêtrière, Paris, France; ^5^CarMeN Laboratory, INSERM, INRA, Université Claude Bernard Lyon 1, Pierre-Benite, France; ^6^International University of Applied Sciences, Bad Reichenhall, Germany; ^7^Institute of Nutritional and Food Sciences, University of Bonn, Bonn, Germany

**Keywords:** gut microbiota, spaceflight, metabolic health, musculoskeletal system, immune system, short-chain fatty acids, astronauts, circadian rhythms

## Abstract

The upcoming exploration missions will imply a much longer duration than any of the missions flown so far. In these missions, physiological adaptation to the new environment leads to changes in different body systems, such as the cardiovascular and musculoskeletal systems, metabolic and neurobehavioral health and immune function. To keep space travelers healthy on their trip to Moon, Mars and beyond and their return to Earth, a variety of countermeasures need to be provided to maintain body functionality. From research on the International Space Station (ISS) we know today, that for instance prescribing an adequate training regime for each individual with the devices available in the respective spacecraft is still a challenge. Nutrient supply is not yet optimal and must be optimized in exploration missions. Food intake is intrinsically linked to changes in the gut microbiome composition. Most of the microbes that inhabit our body supply ecosystem benefit to the host-microbe system, including production of important resources, bioconversion of nutrients, and protection against pathogenic microbes. The gut microbiome has also the ability to signal the host, regulating the processes of energy storage and appetite perception, and influencing immune and neurobehavioral function. The composition and functionality of the microbiome most likely changes during spaceflight. Supporting a healthy microbiome by respective measures in space travelers might maintain their health during the mission but also support rehabilitation when being back on Earth. In this review we are summarizing the changes in the gut microbiome observed in spaceflight and analog models, focusing particularly on the effects on metabolism, the musculoskeletal and immune systems and neurobehavioral disorders. Since space travelers are healthy volunteers, we focus on the potential of countermeasures based on pre- and probiotics supplements.

## Introduction

When entering microgravity the bodies system starts to adapt to the new environment. Fluid shifts from the lower into the upper part of the body and might alongside other factors, cause changes in gastrointestinal function. In combination with reduced fluid intake often seen in space travelers, this might cause reduced gastrointestinal motility. Gastrointestinal transit time has not been systematically studied during flight, but results from analog studies [rats hindlimb suspension (HU) and human short-term bed rest] show that the transit time was significantly longer than during ambulatory control periods (Lane et al., 1993; Shi et al., 2017). Lowering of mechanical loading leads to muscle breakdown and loss of bone mass (Smith et al., 2005; LeBlanc et al., 2007; Grimm et al., 2016). Data from spaceflights has also shown that most space travelers do not achieve their required energy intake through the on-board rations and typically consume about 75–80% of their daily requirements (Zwart et al., 2014). This is associated with a variety of effects on space travelers ranging from a decrease in cognitive ability to general microgravity-induced physiological responses, such as impaired cardiovascular performance, exacerbated muscle atrophy and diminished immune function (Smith et al., 2014). For shorter spaceflights up to 6 months, these effects could be reversed but when moving on to exploration missions of 1 year and longer, the effects may be much more serious and lead to mission failure, i.e., early return because of malnourishment and consequent diseases or even survival of space travelers as has been seen with some pioneering expeditions on Earth.

Inadequate energy intake has been confirmed by short-term missions (8–14 days) where total energy expenditure (TEE) was analyzed by the double-labeled water method (Lane et al., 1997). While TEE did not change in spaceflight, energy intake of these space travelers decreased leading to negative energy balances and loss in body mass (Lane et al., 1997; Stein et al., 1999b). A common cause of reduced dietary intake during the first days of a mission seems to be space motion sickness (Seddon et al., 1994; Reschke et al., 1998; Lackner and DiZio, 2006). The effects of space motion sickness typically pass after the first several days of flight, but the decreased dietary intake can extend well beyond the first week (Stein et al., 1999b). Inadequate energy intake is associated not only with loss of fat tissue, but also with decreased protein synthesis (in flight), increased protein catabolism (in bed rest), and subsequent loss of lean tissue mass (Stein et al., 1999a). Existing data suggest that systems such as muscle, bone and cardiovascular systems are adversely affected by inadequate energy intake. Studies show that undernutrition, depending on severity, exacerbates the negative effects of bed rest/spaceflight on muscle mass and strength (Biolo et al., 2007), bone mass (Ihle and Loucks, 2004), motor and cognitive function (Phillips, 1994), and the cardiovascular system (Smith et al., 2009; Florian et al., 2015).

Adaptive mechanisms in microgravity lead to an optimal state for the microgravity environment but the intention is that space travelers who fly to Moon or Mars with some level of gravity, are immediately fit for duty. They won’t stay in this environment forever, they will get back to Earth and should be healthy and able – after a short recovery - to live in the 1 G environment. Many countermeasures, mainly different training regimes and modification in nutrient supply, have been tested so far but none of them fully maintains the physiological condition in a 1 G environment.

Food intake is intrinsically linked to the composition and function of the gut microbiome. Recent research provides a growing body of evidence demonstrating that host appetite and food intake are linked to the gut microbiome (Alcock et al., 2014; Fetissov, 2017). Perhaps this is due to the fact that most of the microbes that inhabit our body provide benefits to the entire host-microbe system, including production of important resources, bioconversion of nutrients, and protection against pathogenic microbes (Turroni et al., 2018). The gut microbiome is also acknowledged to be critical in maintaining immunological and neurological homeostasis. However, there may be altered (i.e., dysbiotic) gut microbial patterns, which promote intestinal inflammation and systemic low-grade inflammation. Both in turn may promote the development of several disorders, such as type 2 diabetes. Reduced insulin sensitivity has been demonstrated in various short- and longer-term space missions (Leach and Alexander, 1975; Leach and Rambaut, 1977; Stein et al., 1994; Hughson et al., 2016). Since diet is recognized as a pivotal determinant of gut microbiome composition and function (Zmora et al., 2019), changing general food habits on Earth to space food or the respective space travelers’ selection might – beside other environmental factors – deeply affect the gut microbiome structure and functionality with repercussions on the space traveler’s health. Extending the countermeasure portfolio by supplementing pre- and or probiotics might be of interest to support health maintenance of space travelers on exploration missions.

In this review, we aim to summarize the effects of changes in the gut microbiome seen on Earth and in microgravity, which might affect the health of space travelers during exploration missions. Our main focus is –with respect to the physiological system- changes in the gastrointestinal tract, energy intake imbalance, altered metabolism and satiety impairment, effects on the musculoskeletal and immune system and neurobehavioral health. For potential measures we review the supplementation of pre- and probiotics, since space travelers are usually healthy individuals and other strategies such as fecal microbiota transfer or application of bacterial phages, although both with great future potential in clinical practice and beyond, seem to be out of scope for the present review.

## Gut Microbiome Changes in Spaceflight and Analog Studies

Space travels are typically associated with several stressors, including microgravity, fluid shifts, galactic cosmic radiation (beyond the Earth’s Van Allen Belt), sleep deprivation with alteration of circadian rhythms, sleep quality and performance proficiency and, in general, stressful conditions due to prolonged isolation and confinement, collectively referred to as “the space exposome” (Crucian et al., 2018). Specifically, for a space traveler, the exposome is recognized to include endogenous processes (i.e., neurohumoral regulation, aging processes and changes in metabolism and immune responses), external exposures related to spaceflight (i.e., radiation, microgravity, pathogens, dietary constraints, overloads during launch and landing, constant noise, hypodynamia and hypomagnetic fields), and the extensive and inevitable social and psychological issues. Since the early 1960s, some of these stressors have been shown in both animal and human studies, to promote gut microbiota dysbiosis, which may drive gastrointestinal disease and metabolic imbalances, as well as changes in bacterial physiology in the spaceflight environment and ground-based analog studies (Nickerson et al., 2004; Wilson et al., 2007, 2008; Barrila et al., 2010; Bailey et al., 2011; Castro et al., 2011; Crabbe et al., 2011).

Among the medical events that occurred from 1981 to 1998 on space shuttle flights, gastrointestinal problems accounted for 8% and rank third after space adaptation syndrome (42%) and neurosensory alterations (17%) (Hamm et al., 2000). The incidence of the types of illness seen during spaceflights is comparable to that observed during extended submarine missions with high rates of occurrence of gastrointestinal diseases and infections. Literature reporting effects of long-duration spaceflight on the gastrointestinal system is still limited, although space motion sickness is associated with transient decrease in normal gastric myoelectrical activity and delayed gastric emptying (Muth, 2006). Diarrhea related to overmedication for constipation has also been reported and treated according to current practices on Earth (Hamm et al., 2000). However, problems to which the gastrointestinal tract is particularly prone are infection and inflammation (Brown, 2015). In this context, the gut microbiome may play a critical role being able to exert a barrier effect against potential enteropathogens, promote the integrity of the epithelial barrier and influence immune function. Also in light of its additional extra-intestinal effects, such as those involved in metabolic and neurological homeostasis (through the gut-brain axis), the numerous disturbances in spaceflight have a strong potential to impair not only gastrointestinal homeostasis but all related symptoms (illness, immune decline, organ malfunction, muscular dystrophy, response to medications and stress).

The gut microbiome is permanently under the influence of endogenous and exogenous variables. Specific spaceflight cues such as exposure to radiation, changes in circadian rhythms and light–dark cycles, drug intake, confinement, intense exercise and microgravity, might impact the composition and functions of the microbiome as a result of continual exposure, as suggested by ground-based literature in both animals and humans (Green et al., 2008; Clark and Mach, 2017; Kaczmarek et al., 2017; Doestzada et al., 2018; Gerassy-Vainberg et al., 2018; Rothschild et al., 2018; Dong et al., 2019). Preserving eubiosis requires a definition of a healthy microbiome, which is the topic of intense research (Backhed et al., 2012; Lloyd-Price et al., 2016; He et al., 2018). High microbiome richness and diversity are generally considered as a recurrent pattern of a healthy gut ecosystem, and, consequently, a marker of stability and resilience to perturbation (Backhed et al., 2012) but there is still no consensus on the actual health-related values (Proctor, 2019) (richness is defined as the total number of bacterial species in a gut microbiome; diversity refers to the number of different species and how evenly they are distributed in a given microbiome). In many cases, decreased microbial richness will be accompanied by metabolic shifts that might be a more suitable read-out of impaired homeostasis (or intervention success) than richness or diversity. Indeed, neither microbial taxonomy (as obtained by 16S rRNA gene-based sequencing) nor the repertoire of microbial genes (as profiled by shotgun metagenomics) actually provide direct insight into active microbial functions, which will impact on the host physiology (Dorrestein et al., 2014). The identification of a core set of microbiome-produced or derived metabolites universally present in healthy individuals who lack overt disease phenotypes, under the hypothesis that alterations in their levels would indicate dysbiosis, would help rationalize preventive/therapeutic personalized countermeasures to strengthen/restore microbiome resilience to deep space exploration.

The gut microbiome of space travelers has been monitored since the early 1970s, although with different techniques over the years, mostly culture-dependent at the beginning while based on advanced omics technologies in recent years. Even the perspectives have changed over time, from the monitoring of microbial health hazards to the detection of microbiota dysbiosis and early testing of manipulation strategies toward a health-promoting layout. The progressive development of sequencing technologies has allowed researchers, since the 2000s, to explore in depth the compositional and functional structure of the gut microbiome, the possible exchange of microbiota within the crew or with the environments and its dynamics during space missions. However, it should be emphasized that to date only a few studies on the gut microbiome from spaceflight have been published so caution must be taken when interpreting the findings discussed below. Data on real missions are available from experiments in mice flown for 37 days on the ISS (Jiang et al., 2019) as well as 13 days aboard Space Shuttle Atlantis STS-135, and confirmed a higher abundance of Clostridiales and fewer Lactobacillales, in line with previous findings from Lencner et al. (1984). Mice flown for 37 days onboard ISS also demonstrated “unchanged richness of microbial community, an altered community structure and an elevated Firmicutes-to-Bacteroidetes ratio” (Jiang et al., 2019). These observations in mice are comparable with the data from a recent study carried out in twin astronauts (Garrett-Bakelman et al., 2019).

More recently, the Astronauts’ Microbiome project has been specifically designed to study the impact of long-term space travel with all its relevant aspects (in terms of microgravity, g-forces, radiation and anxiety) on the microbiome of crew members and surrounding ISS environment, and the consequences on human health. Skin, saliva, nostril and fecal samples were collected from 9 astronauts prior to launch, during and after 6-month and 1-year missions, along with ISS surface swabs taken from module locations used every day, such as sleeping quarters, exercise equipment and handled microphone. In parallel, innate and adaptive immune responses were evaluated by sampling saliva and blood, and astronauts were asked to fill in an Environmental Health and Hygiene survey to recover metadata on subject health and hygiene as well as environmental factors, such as temperature and humidity. The data demonstrates that the microbiome composition of the gastrointestinal tract, skin, nose and tongue changed in microgravity and became more similar between astronauts (Voorhies et al., 2019). However, as the authors state, it is not clear whether these microbiota alterations represent a risk to the health of astronauts. With specific regard to the gut microbiota, they report space-associated increases in the relative abundance of the beneficial butyrate producer *Faecalibacterium*, but also of *Parasutterella*, which has previously been associated with chronic intestinal inflammation. Furthermore, they found reduced proportions of genera with anti-inflammatory properties, such as *Akkermansia*, possibly contributing to the moderate increase in the inflammatory immune response observed in the crew during spaceflight. The authors therefore suggest the implementation in space of prebiotics or next-generation probiotics, such as *Akkermansia*, to reduce the risk of diseases associated with chronic inflammatory responses.

By sampling twin astronauts, one of whom stayed on the ISS for 1 year while the other on Earth, the Twins Study provided a unique opportunity to understand the health impact of long-duration spaceflight while controlling for genetics (Garrett-Bakelman et al., 2019). Through multidimensional, longitudinal assays, changes in physiological, telomeric, transcriptomic, epigenetic, proteomic, metabolomic, immune, microbiomic, cardiovascular, vision-related and cognitive parameters were assessed. Most of the biological and human health variables returned to baseline after mission but some changes persisted, including gene expression levels, increased DNA damage and number of short telomeres, and attenuated cognitive function. With specific regard to the gut microbiome, notwithstanding individual features and personalized temporal variations, more changes in microbial community composition and function were found during the flight period, with a spaceflight-specific increase in the Firmicutes-to-Bacteroidetes ratio, not persisting upon return to Earth. While in space, changes in small-molecule markers of microbial metabolism were also observed, with particularly low levels of metabolites with anti-inflammatory activity (such as 3-indole propionic acid). On the other hand, as anticipated above, the microbiome diversity remained substantially unchanged. A marked impact on the composition and functionality of the gut microbiome, without compromising individual specificity, has also recently been observed in the short term (15 and 35 days) in two spaceflight missions successfully completed from China (Liu Z. et al., 2020). In particular, according to the authors, *Bacteroides* abundance increased, consistent with simulated space environment tests, while that of the probiotic taxa *Lactobacillus* and *Bifidobacterium* decreased, possibly affecting host immune function. Furthermore, there were fluctuations in antibiotic resistance genes, mobile genetic elements, virulence genes and genes related to biofilm formation worthy of further attention, as they seem to suggest increased virulence potential and possibility of infection by opportunistic pathogens or pathobionts of the gut microbiota in space missions. Such mechanisms may parallel viral activation and infection by opportunistic pathogens as shown through the shedding of viral DNA in the body fluids of astronauts associated to the duration of spaceflight (Rooney et al., 2019).

In recent years, a number of papers have been published on space simulations, involving both animal models and human subjects (Casero et al., 2017; Turroni et al., 2017; Hao et al., 2018; Dong et al., 2019; Jiang et al., 2019). For instance, by using a mouse model for exposure to high linear energy transfer ionizing radiation (^16^O), Casero et al. (2017) reported a pro-inflammatory dysbiotic profile (including decreased proportions of *Bifidobacterium*, *Lactobacillus* and *Clostridiaceae* members) with increased levels of metabolites mechanistically linked to gut epithelial loss (e.g., *N*-acetyl-L-citrulline) that persisted at least 30 days after a single exposure to radiation. However, it should be pointed out that ^16^O exposures were performed at high dose rates, not actually reflecting the continuous low dose rate exposure occurring in space.

In the framework of ground-based analog studies, such as MARS500, a 520-day simulation study conducted at the Institute of Biomedical Problems of the Russian Academy of Sciences in Moscow (Russia), Turroni et al. (2017) explored the temporal dynamics of the gut microbiota of six crew members across the entire duration of the mission, including the period before entering isolation modules and after the return to regular life, up to 6 months later. Probably the most interesting fact is that some microbiota components followed similar trajectories (i.e., increased relative abundance of *Bacteroides* spp. in the very first stage of the mission and decreased proportions of some short-chain fatty acid (SCFA) producers, especially *Faecalibacterium prausnitzii*, around about 1 year of confinement), regardless of the baseline profile, that paralleled major alterations at psychological (dominance of negative feelings and increased salivary cortisol), intestinal health (positivity to the calprotectin test), and immune function level (higher lymphocyte numbers and immune responses), thus potentially serving as red flags for the space traveler’s health, to identify early warning periods and promptly adopt the necessary countermeasures. A parallel experiment, MICHA (MIcrobial ecology of Confined Habitats and humAn health), has instead drawn attention to the microbiology of the environments where space travelers dwell, identifying areas with human activity as hotspots for dispersal and accumulation of crew’s microorganisms, especially of potential pathogenic, stress-tolerant or mobile element-bearing microbes (Schwendner et al., 2017).

More recently, ground-based space simulations have provided intriguing (although not entirely unequivocal) insights into the possibility of maintaining a eubiotic gut microbiome layout (poor in potential pathobionts while rich in health-promoting SCFA producers) through a bioregenerative life-support system (BLSS), i.e., a confined, self-sustained artificial ecosystem to biologically regenerate O_2_, food, water and other basic living necessities (Hao et al., 2018; Chen et al., 2020). In short, the crewmembers followed a fixed schedule that included contact with plants for several hours a day and a high-plant high-fiber diet. Although with a certain individuality and some conflicting data, probably related to the different duration of cohabitation (60 vs. 105 days), both studies have highlighted an impact on the gut microbiome, which varies from an increase in richness and diversity, to an increased relative abundance of some SCFA producers and reduced proportions of potential pathogens. Despite the difficulties in translating this approach into real space missions, these studies are worthwhile as they stress the importance of dietary guidance, with high fiber intake, as a potential means of balancing the gut microbiome and maintaining the space traveler’s health in the long term.

## Gut Microbiome and Metabolic Health

Low-caloric intake with inadequate intake of micronutrients is generally associated with increased inflammation and oxidative stress, and could have possible repercussions on the functioning of the immune system (Bergouignan et al., 2016; Crucian et al., 2018). Although the space travelers’ diet cannot yet be defined as optimized, considerable progress has been made since then, with the average caloric intake having been significantly increasing in recent years. However, it remains a fact that during spaceflights astronauts and cosmonauts eat less than on Earth, probably for several reasons, including but not limited to cultural habits (but the international coordination imposed by ISS is changing this aspect), the palatability of foods (still not comparable to what is available on Earth), space motion sickness, changes in light-dark cycle and appetite-regulating hormones, and, in general, stress (Laurens et al., 2019). Though the reasons are not entirely clear, impaired glucose and lipid metabolism, with insulin resistance and glucose intolerance, are also frequently observed in both spaceflights and ground-based microgravity analogs, representing a serious concern for the general health of space travelers (Tobin et al., 2002; Hughson et al., 2016; Wang Y. et al., 2019).

As a countermeasure, providing the crewmembers with balanced diets, optimized to reduce nutrient deficiency, along with functional foods/bioactive compounds might help improve energy supply and prevent nutritional imbalances, counteracting the potential downstream dysregulation of the immune system. Such diets should be rich in fibers, possibly delivered through Biological Life Support Systems (BLSSs), as non-digestible carbohydrates are well known to exert multiple benefits on human health, mediated by the gut microbiome fermentation in SCFAs (Kolodziejczyk et al., 2019). Acting as signaling molecules (e.g., through G protein-coupled receptor binding or inhibition of histone deacetylase), these microbial byproducts are recognized to be variously involved in energy extraction and storage or, more generally, in maintaining metabolic homeostasis, with some of them, especially butyrate, being potent immune modulators (Koh et al., 2016; Makki et al., 2018). For example, they have been shown to improve glucose and lipid metabolism, by inducing intestinal gluconeogenesis and production of glucagon-like peptide-1 (GLP-1) and peptide YY (PYY), and regulating lipolysis/lipid incorporation (Cani et al., 2009). Specifically, butyrate and propionate have been reported, in rats, to trigger intestinal gluconeogenesis gene expression through complementary mechanisms, i.e., by increasing the cAMP concentration in colonocytes for butyrate, and through a gut-brain neural circuit involving the GPR41 receptor for propionate, which can itself be converted into glucose. Propionate along with acetate is also a potent activator of GPR43, resulting in the secretion of GLP-1 and PYY. Acetate has been found to be responsible for the anti-lipolytic properties of SCFAs. Acting as a preferred energy source for colonocytes, butyrate is fundamental to preserve the integrity of the epithelium and maintain anaerobiosis in the gut lumen, thus limiting aerobic expansion of opportunistic pathogens. Similarly to acetate and propionate, it retains the potential to control distant organs by activating hormonal and nervous systems, and probably represents the SCFA to which most of the beneficial effects are attributed (please see also the following sections). SCFAs have also been shown to control the production of the anorexigenic hormone leptin in adipocytes, which is well known to play a central role in human basal metabolism, regulating glucose homeostasis, insulin and GLP-1 secretion, and appetite (see for a review Turroni et al., 2018). With specific regard to appetite control, a microbiome–host integrative homeostatic model has recently been proposed, according to which gut microbes may regulate intestinal release of satiety hormones and directly activate central appetite pathways mainly through molecular mimicry of microbial antigens (e.g., caseinolytic peptidase B from *Escherichia coli*) that cross-react with hunger and satiety hormones (Fetissov, 2017). Other plausible biological mechanisms involved in microbiome control of eating behavior include manipulation of reward pathways, production of mood-altering toxins, changes to taste receptors and hijacking of neurotransmission via the vagus nerve (see for a review Alcock et al., 2014).

A healthy-like gut microbiome profile, capable of producing SCFAs, especially butyrate, while low in pathobionts, could therefore be decisive in ensuring a fine regulation of host energy metabolism, by maintaining a balance between orexigenic and anorexigenic signals, especially in long-term missions, when energy deficits are no longer tolerable. It should also be remembered that microbial metabolism of fiber has additional, SCFA-independent beneficial effects, ranging from increased availability of ferulic acid and macro/micronutrients released when fibers are metabolized, to the regulation of bile acids levels (Makki et al., 2018). Of note, the use of fibers for preventive, therapeutic application has shown variable results in human intervention studies (Martinez et al., 2010; Davis et al., 2011). Such conflicting phenotypes may result from both the nature of the fibers as well as the individual basal composition in microbial enzymes supporting fibers digestion (Kovatcheva-Datchary et al., 2015; Chen T. et al., 2017). Thus, personalized nutritional approaches that evaluate space travelers’ responses will have to be anticipated on the ground, based on enterotype/metabolotype identifications. In a blinded randomized controlled dietary intervention study, such an approach successfully identified microbiome-based features underlying glycemic responses (Zeevi et al., 2015).

In addition to the design of balanced diets enriched in prebiotics, probiotics-based countermeasures could also be taken into consideration. For example, in a recent spaceflight analog study based on HU mouse model, Wang Y. et al. (2019) have shown that the supplementation of *Bifidobacterium* spp. suppressed endotoxemia and liver inflammation, and improved glucose tolerance. It is also worth noting that the relative abundance of *Akkermansia muciniphila* [found on Earth to be associated with improved metabolic health (Everard et al., 2013) and whose supplementation was recently demonstrated to improve several metabolic parameters in the first human proof-of-concept exploratory study (Depommier et al., 2019)] was significantly reduced in HU mice over time, thus paving the way for its possible use in space missions as well. As for appetite, most of the literature is consistent in reporting positive associations between probiotics (mainly lactobacilli and bifidobacteria) and increased satiety (Falcinelli et al., 2018), while one of the main goals is to have space travelers eating more with adequate macro- and micronutrient supply. Beside that, many reports, based on both analog and spaceflight studies, suggest reduced levels of SCFAs (Turroni et al., 2017; Voorhies et al., 2019). Without prejudice to the usefulness of traditional probiotics in improving several aspects of host physiology (as discussed in other sections of the present review), the available data also support the possible administration to space travelers of SCFA-producing next-generation probiotics (also called live biotherapeutics – O’Toole et al., 2017), such as *Faecalibacterium*, *Roseburia*, etc. However, it should be stressed that many of these novel probiotic candidates are still in the very early stages of the mechanistic investigation and are currently not available on the market.

Several space missions have evidenced relationships between sleep quality, circadian rhythm stability, and performance proficiency in both ground-based simulations and space mission studies. Transcriptomic profiling studies have shown that about 10 percent of our genes are under circadian control (Ueda et al., 2004). With specific regard to issues related to alterations of circadian rhythms in space travelers, the gut microbiome has recently been proposed as an endogenous circadian organizer, capable of influencing epigenetic, transcriptional and metabolic programming in the whole body, thereby impacting diurnal fluctuations of host physiology and disease susceptibility (Thaiss et al., 2016). While well-scheduled sleep, wake rhythms and meal times can serve as synchronizers (Yamamoto et al., 2015), probiotics or other microbiome-modulating approaches might help mitigate the cumulative effects of sleep and circadian disruption and enhance operational performance. For example, heat-killed *Lactobacillus brevis* SBC8803 has been shown to modulate circadian locomotion and sleep rhythms in rodents, through enhanced intestinal release of serotonin (5-HT) and efferent vagal nerve activity mediated by 5-HT3 receptors, which also resulted in increased appetite (Miyazaki et al., 2014). The research in this field is still in its beginning, largely based on animal models and therefore of limited transferability to the human system, and there is a need to better appreciate the molecular mechanisms of probiotics action if they are to be integrated into spaceflight clinical practice. Of course, one of the main challenges would be the standardization of probiotics used in a universally accepted measurement-based approach that considers personal sensibility (Suez et al., 2019).

## Gut Microbiome and the Musculoskeletal System

The most important changes caused by microgravity after long-term stay in space are bone loss and muscle atrophy that occur mostly in weight-bearing bones and their associated skeletal muscles. Regarding bones, the main deleterious mechanism appeared to be an increased bone resorption activity during spaceflight (Smith et al., 2012; Bloomfield et al., 2016). Recent evidence suggested that the gut microbiome might be a novel actor to consider in the regulation of bone physiology in health and disease. The consequences of gut dysbiosis on bone tissue involve complex mechanisms including alteration in minerals and vitamin intestinal absorption and, importantly, modulation of immunity and inflammation. It has recently been demonstrated that activation of inflammation and innate immunity by gut microbiota components increases the production of TNFα and the osteoclastogenic factor RANKL (receptor activator of nuclear factor kappa-B ligand) in bone, and as a consequence, promotes bone loss that can be estimated by a reduction in cortical bone thickness (Ohlsson et al., 2017; Ibanez et al., 2019). This effect of the microbiome on bone is supposed to be dependent on bacterial peptidoglycan sensing by the NOD receptors NOD1 and NOD2 (Ohlsson et al., 2017). Some other potential mechanisms linking the gut microbiome and bone physiology have recently emerged from studies using germ-free mice supplemented with specific bacterial strains. While not representing a model of bone loss, germ-free mice are characterized by reduced body and bone growth when compared to conventional counterparts. Fascinatingly, gut colonization of germ-free infant mice with a specific strain of *Lactobacillus plantarum* (*L. plantarum* WJL) was able to recapitulate juvenile growth, including radial and longitudinal bone growth (Schwarzer et al., 2016). The proposed mechanism for this effect is a strain-dependent stimulation of the somatotrophic axis and the production of IGF-1. The authors also discussed that optimization of enterocyte nutrient uptake and SCFA production may explain the modulation of serum IGF-1 levels (Poinsot et al., 2018). The potential effects of these bacterial strains on osteoporosis or protection against bone loss have not been reported so far.

Considering these relationships between the gut microbiome and bone homeostasis, probiotics are now suggested as an attractive strategy to protect against bone loss (Pacifici, 2018). Supplementing probiotics has been tested as a potential measure to improve the musculoskeletal system. Probiotics, for instance, can modulate the synthesis of vitamins and co-enzymes that are required for matrix formation and bone growth including vitamin D, K, C and folate. Furthermore, by producing SCFAs, they reduce intestinal tract pH and consequently increase mineral absorption (Collins et al., 2017). Accordingly, an increased production of SCFAs in the gut has been correlated with increased calcium absorption and increased bone density and strength in animal models (Chen Y. C. et al., 2017). Finally, the effects of probiotics in enhancing the epithelial barrier function, associated with the regulation of the immune response, have also been suggested as possible contributors to their beneficial effects on bone health (Pacifici, 2018).

Studies in healthy mice and those with mild inflammation suggest that the oral administration of *Lactobacillus reuteri* ATCC PTA 6475 may, in a gender-dependent manner and with different time response, lead to significant increases in femoral and vertebral trabecular bone density, trabecular number, trabecular thickness, mineral apposition rate, bone mineral content and bone mineral density (BMD) (McCabe et al., 2013; Collins et al., 2017). Similar results have been shown in ovariectomized mice, i.e., the classical model of bone loss and osteoporosis due to estrogen deficiency (Zhao, 2013). Hence, supplementation with either *L. reuteri* ATCC PTA 6475 or the commercially available VSL#3 preparation (including *Bifidobacterium*, *Lactobacillus* and *Streptococcus* strains) decreased osteoclastogenesis and bone resorption (Britton et al., 2014; Li et al., 2016). Considering that the response to probiotic supplementation might involve a possible inhibition of inflammation, the findings indicate that in a pro-inflammatory state, probiotics reduce bone resorption and potentiate bone formation, two processes that are classically affected by inflammation (Pacifici, 2018).

Very few clinical studies on the effects of probiotic supplementation focusing on bone loss prevention in humans have been published to date. Nilsson et al. (2018) performed a double-blind, placebo-controlled study involving 70 women (75–80 years old) with low BMD supplementing *L. reuteri* or placebo. After 12 months, women in the *L. reuteri* group showed a lower loss of volumetric BMD (vBMD) compared to placebo. However, none of the secondary bone variable outcomes (BMD measured at the hip and spine; trabecular bone volume fraction; cortical vBMD and cortical thickness) was significantly affected although there was a trend for a beneficial effect for each of them. Biomarkers of bone turnover or inflammation status were unchanged (Nilsson et al., 2018).

Muscles and bones together the “forces” and “rods” of the articulation levers, are the mechanical pillars of mobility. Their development and homeostasis are intimately coordinated by the so-called mechanostat, which couples muscle activity to bone (re)modeling (Frost, 1998). Fascinatingly, it has been demonstrated in the past 10 years that skeletal muscles and bones communicate with each other through the release of hormones called myokines and osteokines, respectively (Brotto and Bonewald, 2015). In many pathophysiological situations, such as aging, immobilization, estrogen deficiency and also microgravity, there is a parallel loss of bones and muscles, suggesting common deleterious mechanisms. While this is clearly demonstrated for inflammation, even subclinical, with TNFα and several pro-inflammatory cytokines (such as IL-17) able to induce both bone loss and skeletal muscle atrophy, additional mechanisms are more specifically involved in the reduction of skeletal muscle mass and strength, such as reduced contraction activity, insulin resistance and low availability of energy fuel substrate or low level of FGF-1 (fibroblast growth factor-1) (Haran et al., 2012; Deutz et al., 2014). As summarized above for bones, there is a growing number of publications pointing to a relationship between gut microbiome and skeletal muscle physiology.

The first evidence arose from observations of changes in the microbiome composition with physical activity, both in animal models and in humans. Several studies in rodents have shown that exercise is associated with higher microbiome diversity and regulation of intestinal integrity and inflammation (Campbell et al., 2016). Some reports also showed exercise-induced changes in the gut microbiome in humans (Pedersini et al., 2020). Bressa et al. (2017) found that several health-promoting bacterial taxa (such as the SCFA producers *Faecalibacterium* and *Roseburia* and the mucin degrader *Akkermansia*) were significantly over-represented in fecal samples of women with an active lifestyle when compared to sedentary age-matched women. Until now, there are very little studies reporting the association between the gut microbiome composition and muscle in situation of muscle atrophy or sarcopenia, while it is well described that the classical consequences of gut microbiome dysbiosis, such as increased circulating levels of lipopolysaccharides, TNFα or other pro-inflammatory cytokines, are able to affect muscle protein synthesis, mitochondrial function in myotubes and skeletal muscle metabolism (Ticinesi et al., 2017; Grosicki et al., 2018).

Interestingly, several compounds and metabolites produced or modified by intestinal bacteria can enter the systemic circulation and affect skeletal muscle biology and function, such as vitamin B_12_, folate or amino acids (like tryptophan), representing critical factors or substrates for muscle protein anabolism (LeBlanc et al., 2013). Other important gut microbiome-derived compounds able to affect skeletal muscles are SCFAs. It has been demonstrated that SCFAs can directly act on skeletal muscle cells, modulating glucose uptake and metabolism, promoting insulin sensitivity (Kimura et al., 2014) and potentially affecting mitochondrial biogenesis through activation of the NAD-dependent deacetylase sirtuin-1 (SIRT1) pathway (Ticinesi et al., 2017). Among SCFAs, butyrate was shown to increase ATP production and improve the metabolic efficiency of myofibers (Leonel and Alvarez-Leite, 2012). In aged mice, the administration of butyrate prevents muscle mass loss (Walsh et al., 2015).

Few studies have evaluated the effects of probiotics and the modulation of the gut microbiome on muscle mass and function. One of the first studies in rodents was in a leukemic mouse model in which Bindels et al. (2012) found a marked gut dysbiosis characterized by selective reduction of *Lactobacillus* spp. associated with muscle cachexia. To restore *Lactobacillus* levels, the authors treated the mice with a probiotic combination of *L. reuteri* 100-23 and *Lactobacillus gasseri* 311476, added to the drinking water for 2 weeks. This treatment was associated with increased tibialis anterior muscle mass and decreased expression of atrogenes in the muscle (MuRF1 and Atrogin-1), as well as decreased serum levels of inflammatory markers (Bindels et al., 2012). More recently, it has also been found in different mouse models of cancer that the administration of *L. reuteri* ATCC PTA 6475 in drinking water can prevent the development of cachexia (Varian et al., 2016). Interestingly, in this study, the authors demonstrated that probiotic supplementation can also protect wild-type mice from age-associated sarcopenia, through a mechanism dependent on the transcription factor Forkhead Box N1 (Varian et al., 2016). Another strain of *Lactobacillus*, *L. plantarum* TWK10, has recently been demonstrated to increase lean mass and improve muscle function (grip strength and swim time tests) in healthy young mice, after oral administration for 6 weeks (Chen et al., 2016). Taken together, these different studies, although in murine model, suggest a possible link between *Lactobacillus* species and skeletal muscle mass and strength that would support further investigation in humans.

To our knowledge, there is no published clinical trial to date testing directly the effects of probiotics supplementation on muscle parameters in humans with muscle atrophy or cachexia. A recent interesting intervention study with older patients involved the administration for 13 weeks of a prebiotic formulation containing fructooligosaccharides and inulin in a randomized controlled trial with 60 volunteers (Buigues et al., 2016). In the treatment group, the subjects experienced a significant improvement in muscle function as estimated by exhaustion and handgrip strength tests (Buigues et al., 2016), supporting the concept that the modulation of the gut microbiome could affect muscle function, muscle strength and possibly muscle mass.

## Gut Microbiome and Neurobehavioral Disorders: Potential Use of Pro/Psychobiotics

Another well-known threat to the success of space missions is the degradation of psychomotor functions and neurocognitive performance, occurring as a result of a multitude of mission-related environmental and psychosocial stressors (De la Torre et al., 2012; De la Torre, 2014). In light of the well-established bidirectional interactions between the gut microbiome and the brain (i.e., the gut-brain axis) (Palma et al., 2020), strategies aimed at maintaining a healthy microbiome might also be helpful in mitigating unwanted neurobehavioral effects. The gut microbiome has indeed been reported to influence, among others, stress physiology and psychology, mood, cognition, and behavior. The bidirectional gut/brain communication occurs directly and indirectly via the central and enteric nervous systems, the vagus nerve, the endocrine and immunoinflammatory systems, and through the modulation of neurotransmitters (Mazzoli and Pessione, 2016; Mittal et al., 2017; Baj et al., 2019). Moreover, gut microbes can themselves produce neuroactive compounds, such as SCFAs and tryptophan metabolites, neurotransmitters (e.g., gamma-aminobutyric acid – GABA, and nitric oxide), hormones or neurotoxic metabolites (i.e., D-lactic acid and ammonia) (Galland, 2014).

Although most of the research concerning intestinal microbiome and mental health is based on rodent studies, human studies have provided preliminary evidence that orally administered probiotics may support mental health (reviewed in Romijn and Rucklidge, 2015; Reis et al., 2018). However, since not all probiotics may be beneficial in all conditions and for all individuals (Romijn and Rucklidge, 2015; Reis et al., 2018; Suez et al., 2018; Zmora et al., 2018), selection of appropriate strains based on the baseline microbiome features and the desired clinical outcome is essential. Specifically, it has been demonstrated that probiotics can modulate the production and release of neuroactive substances. For instance, *Lactobacillus* and *Bifidobacterium* species secrete GABA, *Bifidobacterium infantis* may increase levels of tryptophan (a 5-HT precursor), and *Lactobacillus acidophilus* may modulate the expression of cannabinoid receptors (Romijn and Rucklidge, 2015; Suez et al., 2018; Zmora et al., 2018). In randomized, double-blind, placebo-controlled studies, it has also been shown that: (i) *Bifidobacterium* spp. modulate resting neural activity that correlates with enhanced vitality and reduced mental fatigue in healthy volunteers during social stress (Wang H. et al., 2019); (ii) *L. plantarum* decreases kynurenine concentration and improves cognitive functions in patients with major depression (Rudzki et al., 2019); and (iii) *L. plantarum* alleviates stress and anxiety in stressed adults through enhancement of the 5-HT pathway, as established by lower expression of plasma dopamine β-hydroxylase, tyrosine hydroxylase, indoleamine 2,3-dioxygenase and tryptophan 2,3-dioxygenase associated with increased expression of tryptophan hydroxylase-2 and 5-HT6 receptor (Chong et al., 2019). Interestingly, by a large-scale metagenomic study of independent microbiome population cohorts (Flemish Gut Flora Project, *n* = 1,054 and Dutch LifeLines DEEP cohort, *n* = 1,070), Valles-Colomer et al. (2019) assembled the first catalog of the neuroactive potential of the gut microbiome and evaluated its role in quality of life and depression. According to their findings, the butyrate producers *Faecalibacterium* and *Coprococcus* are consistently associated with higher quality of life indicators probably through the production of butyrate as well as of the dopamine metabolite 3,4-dihydroxyphenylacetic acid. *Coprococcus* and *Dialister* spp. were also found to be depleted in depression, even after correcting for the confounding effects of antidepressants. The authors also indicated a potential role of microbial GABA production in depression. The glutamate degradation pathway I (to crotonyl-coenzyme A and acetate) and the GABA synthesis pathway III (GABA shunt pathway), tended to be respectively depleted and increased in participants with depression, thus representing future population-based knowledge and rationally based objective for probiotic choice in clinical studies (Valles-Colomer et al., 2019). SCFAs (acetate, propionate, and butyrate) might also influence psychological functioning via interactions with G protein-coupled receptors or inhibition of histone deacetylases, and exert their effects on the brain via direct humoral effects, indirect hormonal and immune pathways and neural routes (Dalile et al., 2019). For instance, SCFAs maintain intestinal barrier integrity and protect from intestinal inflammation (Lewis et al., 2010). Butyrate, in particular, can enhance intestinal barrier function by regulating the expression of tight junction proteins, mediated by the activation of AMP-activated protein kinase (Peng et al., 2009) and downregulation of claudin 2 expression (Daly and Shirazi-Beechey, 2006). In a rodent model, *Bifidobacterium* alleviates symptom of depression and related microbiota dysbiosis, with improvement of serotonin levels and brain-derived neurotrophic factor (BDNF) concentration in brain (BDNF is essential for neuronal development and survival, synaptic plasticity, and cognitive function), and reduced serum corticosterone level and increased cecal butyrate level, which were significantly and positively correlated with depression-related indexes (Tian et al., 2019). The health effects that bifidobacteria exert can be the result of interactions with the resident gut microbiota (Cani and Van Hul, 2015), as a result of cross-feeding interactions between bifidobacteria and butyrate-producing colon bacteria, such as *F. prausnitzii* (clostridial cluster IV), and *Roseburia* species (clostridial cluster XIVa) (Rivière et al., 2016). Hence, all these taxa (i.e., *Dialister*, *Coprococcus*, *Bifidobacterium*, *Faecalibacterium*, and *Roseburia*) could represent potential leads for psychobiotics, i.e., probiotics capable of conferring mental health benefits (Dinan et al., 2013), and whose utility in space missions deserves dedicated research.

## Gut Microbiome and Immune System Decline

The effects of spaceflight on the immune system have been reported for several decades (Cogoli, 1993; Konstantinova et al., 1995; Sonnenfeld, 1998; Stowe et al., 1999; Sonnenfeld et al., 2003). Overall, all immune populations are affected in number, proportion, generation, and/or function. In general, a decrease in immunity is observed during spaceflight. This results, in particular, in the reactivation of latent herpesviruses such as Varicella-zoster virus (VZV), Epstein-Barr Virus (EBV), and cytomegalovirus (CMV) (Mehta et al., 2013, 2017; Rooney et al., 2019). The causes of these immune changes can be directly related to spaceflight (microgravity, radiation, etc.) or indirectly (microbiome, bone metabolism, nutrition, anxiety, depression, infections, etc.), and can hardly be dissociated in the studies carried out. Most common alterations, such as an increase in white blood cells, granulocytes, and a decrease in NK cells, are generally found in both humans (Stowe et al., 1999; Crucian et al., 2000, 2015) and mouse models (Crucian et al., 2008; Gridley et al., 2009), regardless of flight time. The phagocytic function of these cells is also reduced (Konstantinova et al., 1995; Simpson et al., 2016). The effects of spaceflight on lymphocytes are less clear. The humoral response mediated by B lymphocytes is not well studied. These cells appear to be minimally involved during spaceflight, since their frequency does not change during flight but seems to be reduced on return to Earth (Tascher et al., 2019). No changes in the immunoglobulin repertoire were observed in mice (Ward et al., 2018) and space travelers studies (Stowe et al., 1999; Rykova et al., 2008). The variations observed in T lymphocytes are more complex. Although there is a decrease in their generation (Benjamin et al., 2016), their numbers remain stable (Crucian et al., 2013, 2015). However, several alterations on these cells have been observed: a decrease in intracellular trafficking (Hashemi et al., 1999; Hatton et al., 2002; Tauber et al., 2015), proliferation (Cogoli et al., 1984; Pippia et al., 1996) and function (Hashemi et al., 1999; Crucian et al., 2008; Bradley et al., 2017). Although EBV-specific T lymphocytes increased, their function is reduced (Mehta et al., 2014, 2017; Crucian et al., 2015). In addition, CD8 T lymphocytes have a more mature phenotype (Crucian et al., 2015). This suggests an unsuccessful attempt by the immune system to eliminate the reactivation of latent viruses. Immune cells secrete cytokines to regulate the immune response by activating, inhibiting and recruiting immune sub-populations. The study of cytokines varies considerably depending on the duration of the flight, cell culture systems used and mitogens added to stimulate cytokine secretion. Indeed, the different mitogens used (Concanavalin A, LPS, PMA-ionomycin, anti-CD3) differentially stimulate cytokines in peripheral blood mononuclear cells (PBMCs) (Crucian et al., 2013). In general, the pro-inflammatory cytokine IFNγ, secreted by CD4 and CD8 T lymphocytes, is decreased during and after flight (Crucian et al., 2000, 2008, 2015). Although the level of the anti-inflammatory cytokine IL-10 varies between studies, the IFNγ/IL-10 ratio remains decreased (Crucian et al., 2008), suggesting a shift in favor of the Th1/Th2 response (Mehta et al., 2013). However, the observations made following the stimulation of PBMCs are opposite to the cytokine assays directly on astronauts’ plasma (Crucian et al., 2013; Mehta et al., 2013; Garrett-Bakelman et al., 2019). Interestingly, the production of IL-17, secreted by Th17 cells associated with the gut microbiota, is also altered (Crucian et al., 2013, 2015; Garrett-Bakelman et al., 2019). It is, therefore, necessary to differentiate between the secretory capacity of cytokines by immune cells and the presence of cytokines in plasma. The immune system and the intestinal microbiome are strongly linked and have been widely studied both as a consequence and as a cause of several immunosuppressed human pathologies such as cancer. Indeed, the modulation of the intestinal microbiome enhances immune response of immunotherapies in the anti-cancer response (Vetizou et al., 2015; Routy et al., 2018; Schramm, 2018). During space travel, the intestinal microbiome is also altered, and it is, therefore, difficult to assess whether it is the cause or the consequence of the observed immune changes. These close links must also receive special attention when using pre- and probiotics. Space travel alters the intestinal microbiota and thus the associated metabolic and immune functions. For example, astronauts’ fiber intake is low, which may lead to a decrease in metabolites associated with the intestinal microbiota such as SCFAs. SCFAs play multiple roles in the immune system, acting directly on their target cells, which mainly carry their receptors FFAR2 (GPR43), FFAR3 (GPR41) and GPR109a, and also having histone deacetylase inhibitor (HDACi) activity. Indeed, butyrate, which can also act as a HDACi, has been shown to inhibit pro-inflammatory cytokine expression in both monocytes and macrophages while simultaneously inducing the expression of IL-10. This has been suggested via a mechanism involving the inhibition of NFκB activation (Kim, 2018). SCFAs are also able to control T cells, especially butyrate. For example, butyrate promotes the generation of regulatory T lymphocytes (Tregs) via the FFAR2 receptor (Smith et al., 2013) and HDACi activity (Arpaia et al., 2013; Furusawa et al., 2013; Smith et al., 2013), thus shifting the immune system to a more tolerogenic phenotype. More recently, several studies support the pro-inflammatory effect of SCFAs. Indeed, acetate, propionate, and especially butyrate increase the IFNγ and Granzyme B secretory activity of CD8 T lymphocytes (Balmer et al., 2016; Luu et al., 2018) and reduce IL-17 secretion by Th17 (Luu et al., 2018) via FFAR2&3 and HDACi activity. Butyrate also allows the differentiation of activated CD8 T lymphocytes toward a memory phenotype (Bachem et al., 2019).

It is accepted that intake of fruit and vegetables-derived dietary fiber in astronauts is rather low (Crucian et al., 2018; Makki et al., 2018). Their low consumption on the ISS would result in low SCFA production. Butyrate supplementation increases the secretion of IFNγ by CD8 T lymphocytes (Luu et al., 2018). In humans, it is commonly accepted that a healthy and balanced diet, with a regular intake of dietary fiber through fruits and vegetables, allows the prevention of several diseases with immune deficiencies such as cancer (Wang et al., 2012; Aune et al., 2016), and a better immune response against pathogens (Desai et al., 2016). In view of the effects on immunity, the use of prebiotics and probiotics to stimulate the production of SCFAs would thus increase nutrient and metabolic resources and the eliminatory capacity of lymphocytes, which may limit the re-emission of latent viruses. The twin study revealed modulations of other microbial metabolites belonging to the indole family, aromatic amino acids and secondary bile acids (Garrett-Bakelman et al., 2019), which are also associated with modulation of the immune system. Indoles are synthesized by commensal bacteria from tryptophan, an essential amino acid. Indoles are non-exclusive ligands of AhR receptors expressed by immune cells, and regulate inflammation genes such as FoxP3, IL-10, IL-6, etc., allowing the preservation of intestinal homeostasis (Gao et al., 2018; Kim, 2018). Tryptophan can be metabolized by the microbiota-dependent indole pathway, the partially microbiota-dependent kynurenin pathway and the microbiota-independent serotonin pathway. The metabolism of tryptophan into these three pathways is balanced. A disruption of this balance in one of the pathways is frequently observed in several diseases (Agus et al., 2018). Synthesized by the gut microbiota from primary bile acids, secondary bile acids bind to their receptors TGR5, FXR and PXR and are implicated in several diseases (Schaap et al., 2014; Zhang et al., 2019). Secondary bile acids also play a role on immune cells by interacting with macrophages, CD4 T lymphocytes, T helper (Th1 and Th17) and Tregs, neutrophils, and NK cells, and control the production of pro-inflammatory cytokines, IFNγ, TNFα, IL-17, IL-6 (Cao et al., 2017; Van den Bossche et al., 2017; Fiorucci et al., 2018; Hang et al., 2019; Song et al., 2020). With the exception of tryptophan, the role of aromatic amino acid metabolites is less known on the immune system but they are found to be deregulated in several immune and other diseases (Liu Y. et al., 2020). All these microbial metabolites offer wide approaches in the modulation of the immune system and must be taken into account in the design of future pre- and probiotics. For example, dietary enrichment with fiber/SCFAs, tryptophan or other microbial metabolites has been shown to improve clinical outcomes in several mouse models of diseases (Matt et al., 2018) such as colitis (Islam et al., 2017; Wang et al., 2020). Interestingly, in one of the rare randomized controlled trials (RCTs) that evaluated the effect of 6-week treatment with a prebiotic/probiotic/synbiotic on immune markers in 45 healthy young individuals, the authors reported a reduction in C-reactive protein, IL-6, IL-1β, and TNFα with a more pronounced reduction in the synbiotic group (Rajkumar et al., 2015). Another RCT that evaluated prebiotic/probiotic/synbiotic effects on vaccine responses to influenza vaccination did report enhanced antibodies titers, albeit with substantial heterogeneity (Yeh et al., 2018) thus holding promise for targeting immune response through such strategies.

## Limitations and Next Steps

As discussed in the sections above, there are many current shortcomings on the usability of probiotics in space. Despite encouraging data on their survival and stability in microgravity environments, the studies available on Earth do not actually allow to draw definitive conclusions on their effects on health/reconstitution of microbiomes. The sample size is sometimes inadequate, only a few strains belonging to a few genera are usually used, different methodologies are employed for sequencing and analysis of microbiome data, mechanistic information is often missing and conflicting data are sometimes reported. Furthermore, host and microbiome baseline information is very often not taken into consideration in strain selection but a one-size-fits-all approach is generally pursued (Suez et al., 2019). While the knowledge of the human gut microbiome, accelerated by next-generation sequencing, has extended the range of microorganisms with suggested health benefits (i.e., next-generation probiotics or live biotherapeutics), many of these are still at the very early stage of mechanistic investigation and only proof-of-concept exploratory studies are currently available. Future directions should therefore include changes at different levels, such as conception, research methodology and approach, which should be a precision mechanism-based one, taking into account host and microbiome features (to identify permissive vs. resistant phenotypes toward probiotics colonization, be it transient or persistent) (Suez et al., 2019), including diet.

Similarly, despite the well-recognized benefits of prebiotics on Earth, particularly those resulting from the promotion of SCFA producers (as “ecosystem service providers”), there are still several issues to deal with, such as the complexity of the mutualistic and competitive interactions that are established in the intestine, the microbiome resilience and individuality in the response to the diet, and from a practical point of view, the definition of the exact dose of fiber to be administered to obtain a certain effect and its tolerability. In this regard, a very recent study suggests that discrete dietary fiber structures may be used for precise and predictable manipulation of the gut microbiome and its metabolic functions relevant to health, by specifically directing changes in the SCFA outputs (Deehan et al., 2020). Rational, machine learning or artificial intelligence approaches are strongly advocated by the literature, to predict the effect of a specific dietary component on physiology, by addressing complex datasets of microbiome and host features (Kolodziejczyk et al., 2019).

Based on the recognized benefits of probiotics on gut microbiome and globally on health, their use either added to food or as supplements during spaceflights might be a promising alternative to counteract the dysregulation and health outcomes encountered by space travelers. However, there are still some questions regarding the persistence of the efficacy of pro (or pre-)biotics under microgravity conditions. To test this, Castro-Wallace et al. (2017) have assessed the behavior of the probiotic strain *L. acidophilus* ATCC 4356 in a microgravity environment. They did not observe differences in growth, survival in simulated gastric or small intestinal juices, or in bacterial gene expression in comparison to control cultures, suggesting that the strain will behave similarly during spaceflight and consequently will maintain its beneficial properties (Castro-Wallace et al., 2017). Recently, Sakai et al. (2018) specifically developed a freeze-dried probiotic product for space experiments using the *Lactobacillus casei* Shirota probiotic strain, and tested its stability over 1 month of storage on the ISS. For the study, a SpaceX/Dragon spacecraft for the 8th commercial resupply mission (SpX-8) was used for the launch to the ISS and return of probiotic samples. The absorbed dose rate of the flight sample was 0.26 mGy/day and the dose equivalent rate was 0.52 mSv/day. The authors did not observe differences between the probiotic flight samples and ground controls regarding the profiles of randomly amplified polymorphic DNA, the sequence variant frequency, the carbohydrate fermentative patterns, the reactivity to strain-specific antibody, and the cytokine-inducing ability of *L. casei* Shirota. Evaluation of survival after 6 months showed that the number of viable cells in the probiotic flight samples was around 11 log CFU/g, a value comparable to that of ground controls (Sakai et al., 2018). Although these results are very encouraging, additional mechanistic studies under microgravity and simulated space environment are still needed, especially to directly test the health benefits of probiotics in space. Once the best bacterial strains will be identified and selected, clinical trials or intervention studies in space travelers should be rapidly carried out to validate their potential during long-term stay in space.

## Conclusion

Studies available to date show that the space exposome can strongly influence the gut microbiota of space travelers, with the potential impairment of the homeostatic relationship with the host. In light of the crucial role of intestinal microbes in maintaining metabolic, immunological and neurological health, as well as of muscles and bones, strategies aimed at recovering and preserving a eubiotic microbiota profile might help mitigate the unwanted effects on the space traveler’s body, thus contributing to the success of long-term missions. This could be achieved by optimizing diets to ensure adequate energy and fiber supply for SCFA production, while avoiding nutritional imbalances, as well as by integrating them with prebiotics, bioactive compounds and probiotics for potentially synergistic effects. Aside from prebiotics and bioactive compounds, probiotics, both traditional and next-generation ones, during spaceflights can be postulated as a non-invasive alternative –given that safety is assured- to protect space travelers against altered metabolism, satiety impairment, immune dysregulation, circadian rhythm changes, bone and muscle loss, as well as neurobehavioral disorders. Additional mechanistic studies under microgravity and simulated space environment, but also intervention studies and clinical trials directly in space travelers are needed to support current evidence on pre-, probiotics or combined strategies on Earth, before these microbiota manipulation tools can be integrated into spaceflight clinical practice. The use of prebiotics for the production of SCFAs is currently being investigated for space travel (Matsuda et al., 2019; Akiyama et al., 2020).

## Author Contributions

All authors listed have made a substantial, direct and intellectual contribution to the work, and approved it for publication.

## Conflict of Interest

The authors declare that the research was conducted in the absence of any commercial or financial relationships that could be construed as a potential conflict of interest.
